# Serum markers for prognostic value of EGFR-TKI in lung adenocarcinoma with bone metastases: a retrospective study

**DOI:** 10.7717/peerj.20537

**Published:** 2026-01-14

**Authors:** Jiali Li, Shuting Wang, Zhiyong Deng, Lu Zhang, Min Liu, Yunlei Luo, Yunqiu Guo, Pengjie Liu, Chao Liu

**Affiliations:** 1 Department of Nuclear Medicine, Puer People’s Hospital, Puer, Yunnan, China; 2 Department of Nuclear Medicine, The Third Affiliated Hospital of Kunming Medical University, Kunming Medical College, Kunming, Yunnan, China; 3 Department of Thoracic Surgery,The Third Affiliated Hospital of Kunming Medical University, Kunming Medical College, Kunming, Yunnan, China

**Keywords:** Lung adenocarcinom, Serum markers, EGFR-TKI, Bone metastases, Prognostic prediction

## Abstract

**Background:**

Lung adenocarcinoma is a prevalent malignancy. Mutations in the epidermal growth factor receptor (EGFR) have introduced novel prospects for targeted therapies. However, the status of EGFR mutations alone may be insufficient to fully predict treatment outcomes. To this end, the present research was performed to evaluate the serum markers associated with epidermal growth factor receptor-tyrosine kinase inhibitor (EGFR-TKI) treatment in patients with lung adenocarcinoma and bone metastases, specifically focusing on the prognostic value of EGFR-TKI therapy.

**Methods:**

A retrospective analysis was conducted on 164 patients diagnosed with lung adenocarcinoma and bone metastases at Yunnan Cancer Hospital between January 2019 and December 2020. Clinical and follow-up data were collected, and a Cox regression model was employed to evaluate the combined predictive value of serum markers for survival outcomes.

**Results:**

The findings revealed that variables identified through Cox regression analysis included age, the neutrophil-to-lymphocyte ratio (NLR), carcinoembryonic antigen (CEA), and Cytokeratin 19 fragment (Cyfra21-1), all exhibiting significance levels of *P* < 0.05. The Cox model exhibited a c-index of 0.644, and the calibration curve demonstrated satisfactory performance, indicating the moderate predictive capacity of the model. A nomogram was subsequently constructed to visualize these results.

**Conclusion:**

This research successfully developed a nomogram based on the Cox regression model to predict prognosis and treatment outcomes in patients with lung adenocarcinoma and bone metastases undergoing EGFR-TKI therapy. This facilitates the avoidance of poor treatment outcomes by enabling individualized therapeutic approaches, thereby simplifying the development of the most appropriate treatment plans and ultimately improving patient prognosis.

## Introduction

Lung cancer is currently the second most common cancer globally, accounting for 11.4% of all cancer cases, and is the highest cancer-related cause of mortality worldwide, contributing to approximately 18% of all cancer-related deaths (Sung et al., 2021). Non-small cell lung carcinoma (NSCLC) accounts for 80–85% of all lung cancers, with around 30–40% of NSCLC patients suffering from bone metastases at the initial disease stages (Miyashita et al., 2018; Hsiao et al., 2020). Lung adenocarcinoma, the predominant histological type of NSCLC, exhibits a higher propensity for bone metastases compared to other adenocarcinoma subtypes, with approximately 20–50% of lung adenocarcinoma patients presenting with bone metastases (Decroisette et al., 2011). Bone metastases are frequently associated with pathological fractures, spinal cord compression, hypercalcemia, hyperuricemia, and radiotherapy or surgery, which are collectively defined as skeletal related events (SREs) (Teng et al., 2020). SREs are a major cause of morbidity in patients and contribute to substantial economic burden in the last stage of the disease.

Some special bone-related markers, detectable in the serum, can be used both during osteolytic or osteoblastic bone metastases as well as in clinical and experimental studies. For example, the type I procollagen amino-terminal peptide (PINP) is a typical one that signifies the new bone formation process, while type I collagen carboxy-terminal peptide (CTX) and other markers of bone resorption may at the same time facilitate the diagnosis of tumour-induced bone metastases. The course of bone metastases can be constantly monitored, and, thus, different measurements of both progression-free survival and median survival among patients can be either increased or decreased (Zhu et al., 2021; Wang et al., 2023). In our univariate and multivariate Cox regression analyses, neutrophil-to-lymphocyte ratio (NLR), carcinoembryonic antigen (CEA), and cytokeratin fragment 21-1 (Cyfra21-1) were identified as independent risk factors for overall survival (OS), whereas alkaline phosphatase (ALP) and lactate dehydrogenase (LDH) were not associated with the prognosis or survival outcomes of patients. Previous research has even found other kinds of blood serum among the bone metabolic markers that function as biomarkers for bone metastases. Among these, CEA and Cyfra21-1 were found to show value in cancer diagnosis, monitoring, and assessment of treatment prognosis (Chai et al., 2021). Previous studies have proposed that serum ALP and LDH levels are independent of patient prognosis in NSCLC with bone metastases. These markers are relevant in tumour biology, as they reflect tumour burden and treatment response. A comprehensive examination of serum markers may serve as a robust tool for predicting the outcomes of EGFR-TKI treatment in patients with lung adenocarcinoma and bone metastases.

The purpose of this study was to identify the prognostic value of a combined assessment of serum markers in patients with lung adenocarcinoma and bone metastases undergoing EGFR-TKI treatment and to provide more accurate personalized treatment planning for clinical practices. Investigating the relationship between serum markers and EGFR-TKI therapy in this group of patients may provide insights into potential prognostic factors and facilitate the optimization of management strategies for these patients.

## Methods

### Patients

This analysis was carried out in strict accordance with the principles of the Declaration of Helsinki (revised in 2013) and approved by the Ethics Committee of Yunnan Cancer Hospital (approval number: KYLX 2024005). Inclusion criteria for patients were as follows: (1) diagnosis of lung adenocarcinoma confirmed by histopathology at Yunnan Cancer Hospital between January 1, 2019, and December 31, 2020; (2) age ≥18 years; (3) availability of EGFR gene tests; (4) use of EGFR-TKIs as the first-line treatment; and (5) complete clinical data. Exclusion criteria were: (1) the presence of other primary malignant tumours; (2) unclear mutation site of the EGFR gene test for the primary tumour, resulting in inaccurate targeted therapy information; and (3) absence of critical data. Based on these inclusion and exclusion criteria, a total of 164 patients were ultimately selected for further analysis, along with the variables considered for inclusion. Follow-up survival assessments were conducted every 3 months during the first 2 years and every 6 months thereafter through outpatient and inpatient reviews, as well as telephone inquiries. For patients lost to follow-up, the most recent survival status was determined through contact with family members or local health authorities, and cases with unconfirmed outcomes were censored at the last known follow-up date.

This study retrospectively analyzed the clinical data of patients diagnosed with lung adenocarcinoma and bone metastases *via* pathology and/or imaging who were admitted to Yunnan Cancer Hospital from January 2019 to December 2020. The following variables were collected to explore their predictive value for prognosis: age, gender, ECOG score, smoking status, BMI, American Joint Committee on Cancer (AJCC) T stage, AJCC N stage, EGFR gene mutation type, occurrence of SREs before and after EGFR-TKI treatment (with the post-treatment period defined as ≥6 months), and the levels of ALP, NLR, LDH, Cyfra21-1, and CEA. Serum markers (ALP, NLR, LDH, Cyfra21-1, and CEA) used in the Cox regression analysis were obtained from baseline blood samples collected within 7 days prior to the initiation of EGFR-TKI treatment. These values reflect the patients’ pre-treatment status. Post-treatment values were collected at 6 months following the initiation of EGFR-TKI therapy, used only for longitudinal trend analysis and were not included in the survival model. All serum biomarkers were measured using standardized protocols in the hospital’s certified central laboratory. CEA and Cyfra21-1 levels were determined using electrochemiluminescence immunoassays (ECLIA), while ALP and LDH were measured *via* enzymatic rate assays. NLR was calculated from routine complete blood counts. Quality control procedures followed national clinical laboratory standards.

### Clinical characteristics

OS was defined as the interval from the initial diagnosis of lung adenocarcinoma with bone metastases (not the first diagnosis of lung adenocarcinoma) to the final follow-up or death. Outcome indicators were defined as follows: survival was coded as 1, and non-survival was coded as 0. Additionally, the survival time and status of the patients were recorded, and staging was conducted using the AJCC 8th edition TNM staging system. Patients were classified as non-smokers if they had smoked fewer than 100 cigarettes in their lifetime, whereas smokers were defined as current smokers or individuals who had ceased smoking within 1 year prior to diagnosis. Additionally, for a subset of patients with available records, we collected EGFR-TKI treatment details, including start and end dates, dose adjustment history, treatment duration, early discontinuation, and any major modifications. Although dose ranges were not uniformly documented, treatment consistency and temporal information provided further insight into the clinical context in which our prognostic model was constructed. Early discontinuation was defined as stopping EGFR-TKI within 6 months of initiation.

### Statistical analysis

Data were processed and analyzed using SPSS version 26.0 statistical software (IBM Corp., Armonk, NY, USA). For measured data that did not conform to a normal distribution, results were expressed as the median along with the upper and lower quartiles, and the Wilcoxon signed-rank test was employed for intragroup comparisons. Count data were presented as frequencies (percentages), with the paired chi-square test utilized for intragroup comparisons. A *P*-value of <0.05 was indicative of a statistically significant difference. Furthermore, univariate and multivariate Cox regression analyses were conducted to delve into factors influencing prognosis and survival outcomes. Additionally, R version 4.2.0 software was used to generate line graphs of the Cox regression model and to create time-ROC curves for predicting survival outcomes at 1, 3, and 5 years. This software enabled risk-benefit analysis and calculation of the area under the curve (AUC), sensitivity, and specificity. Calibration curves for 1, 3, and 5 years were made in order to clarify the correctness of model predictions. A 45° reference line was drawn on the calibration plot. The proximity of the calibration curve to this reference line reflects the concordance between predicted probabilities and observed outcomes, with greater alignment indicating enhanced predictive accuracy. The C-index of the model was also estimated, with values above 0.5 indicating a well-fitted model. In conclusion, the decision curve analysis (DCA) was performed for 1, 3, and 5 years to assess the clinical utility of the model in the forecasting of patient surviving outcomes.

## Results

### General data description

This study included a total of 164 cases. There were 65 males (39.6%) and 99 females (60.4%), aged 31–83 years, with a mean age of 67.14 ± 8.38 years, 24 of which were <65 years old (14.6%) and 140 were ≧65 years old (85.4%). T-stage: 28 T1 cases (17.1%), 46 T2 cases (28.0%), 28 T3 cases (17.1%), and 62 T4 cases (37.8%). N-stage: 41 N0 cases (25.0%), 32 N1 cases (19.5%), 17 N2 cases (10.4%), and 74 N3 cases (45.1%). ECOG rating: 46 cases (28.0%) in grade 0, 108 cases (65.9%) in grade 1, nine cases (5.5%) in grade 2, and one case (0.6%) in grade 3. Smoking history: 40 cases (24.4%). Medication use, first-generation nbsp; EGFR-TKI: 123 cases of gefitinib/icotinib (75.0%), second-generation EGFR-TKI: nine cases of afatinib (5.5%); third-generation EGFR-TKI: 32 cases of ametinib/osimertinib/anlotinib (19.5%); 133 cases positive for exon 19 deletion (81.1%); 133 cases positive for exon-21 L858R (81.1%); 116 cases positive for exon-20 T790M (70.7%); 14 cases positive for exon-20 S7681 (8. 5%); 19 cases positive for exon-18 G719X (11.6%); six cases positive for exon-21 L861X (3.7%); and 14 SRE-positive cases (8.5%). Median ALP was 97.00 (75.50, 139.00); median NLR was 3.79 (2.33, 7.36); median LDH was 210.50 (175.25, 284.25); median Cyfra21-1 was 5.90 (3.60, 12.18); and median CEA was 70.93 (23.67, 201.42). The median follow-up duration was 30.5 months (range: 6.1–130.9 months). At the end of follow-up, four patients (2.4%) were censored due to being alive. These data provide sufficient follow-up duration for estimating 1-, 3-, and 5-year overall survival. Details are presented in Table 1.

**Table 1:** General data description.

Indicator	*n*(%)[P50(P25, P75)]
Sex, *n* (%)	
Male	65 (39.6)
Female	99 (60.4)
Age, *n* (%)	
<65	24 (14.6)
≧65	140 (85.4)
T-stage	
1	28 (17.1)
2	46 (28.0)
3	28 (17.1)
4	62 (37.8)
N-stage	
0	41 (25.0)
1	32 (19.5)
2	17 (10.4)
3	74 (45.1)
BMI	22.04 (20.20, 24.22)
ECOG rating	
0	46 (28.0)
1	108 (65.9)
2	9 (5.5)
3	1 (0.6)
Smoking history	
Yes	40 (24.4)
no	124 (75.6)
Medicate	
First-generation EGFR-TKI: gefitinib/icotinib	123 (75.0)
Second-generation EGFR-TKI: afatinib	9 (5.5)
Third-generation EGFR-TKI: Ametinib/Osimertinib/Anlotinib	32 (19.5)
Exon 19 deletion	133 (81.1)
Exon-21 L858R	133 (81.1)
Exon-20 T790M	116 (70.7)
Exon-20 S7681	14 (8.5)
Exon-18 G719X	19 (11.6)
Exon-21 L861X	6 (3.7)
SREs	14 (8.5)
ALP	97.00 (75.50, 139.00)
NLR	3.79 (2.33, 7.36)
LDH	210.50 (175.25, 284.25)
Cyfra21-1	5.90 (3.60, 12.18)
CEA	70.93 (23.67, 201.42)

Among the 76 patients with EGFR-TKI treatment records, 74 had documented start and end dates. The median treatment duration was 266.5 days (interquartile range: 7.5–549 days). A total of 9.1% (7/76) of patients discontinued EGFR-TKI therapy within 6 months. Additionally, 17 patients experienced major treatment modifications, such as switching from gefitinib to osimertinib or adding anlotinib. These changes reflect real-world management adjustments in response to disease progression. Regarding co-treatments, 21.1% of patients received chemotherapy, radiotherapy, or surgery prior to EGFR-TKI initiation, and 59.2% received such treatments following EGFR-TKI therapy. These therapies were typically administered after progression or as part of comprehensive care.

### Comparison of markers before and after treatment

A paired chi-square test and Wilcoxon signed-rank test revealed that the positive rate of SREs significantly increased after the treatment period (*P* < 0.05). Additionally, the levels of ALP, NLR, LDH, Cyfra21-1, and CEA all showed a significant decrease following treatment (*P* < 0.05). Refer to Table 2.

**Table 2:** Comparison of markers before and after treatment.

Indicator	Before treatment (*n* = 164)	After treatment (*n* = 164)	χ^2^/*z* value	*P* value
SREs, *n* (%)	14 (8.5)	31 (18.9)	13.474	<0.001
ALP	97.00 (75.50, 139.00)	76.50 (60.50, 105.00)	−6.732	<0.001
NLR	3.25 (2.08, 4.74)	2.35 (1.60, 3.46)	−4.598	<0.001
LDH	202.00 (171.25, 262.25)	201.00 (174.00, 239.75)	−2.048	0.041
Cyfra21-1	5.40 (3.50, 9.40)	3.20 (2.20, 6.15)	−4.855	<0.001
CEA	25.88 (7.01, 124.55)	11.52 (3.02, 56.02)	−4.151	<0.001

### Analysis of factors influencing Cox regression of prognostic OS outcomes

#### Univariate Cox regression analysis of prognostic OS outcomes

Cox regression analysis was performed with OS status as the dependent variable. The independent variables included sex, age, T-stage, N-stage, BMI, ECOG rating, smoking history, medication use, exon 19 deletion, exon-21 L858R, exon-20 T790M, exon-2 0 S7681, exon-18 G719X, exon-21 L861X, SREs, ALP, NLR, LDH, Cyfra21-1, and CEA. Variable assignments are detailed in Table 3. The analysis identified age $\geqq$65, positive exon 19 deletion, positive exon-21 L858R, positive exon-20 T790M, higher NLR, higher LDH, higher Cyfra21-1, higher CEA, and N2 N-stage as risk factors associated with OS outcomes. See Table 4.

**Table 3:** Variable assignment table.

Variable	Assignment method
Sex	Female value = 0, male value = 1
Age	<65 years old is assigned a value of 0, ≧65 years old is assigned a value of 1
T-stage	T1 is assigned the value 1, T2 is assigned the value 2, T3 is assigned the value 3, and T4 is assigned the value 4.
N-stage	N0 is assigned the value 0, N1 is assigned the value 1, N2 is assigned the value 2, and N3 is assigned the value 3.
BMI	Enter the original value.
ECOG rating	0 assignment = 0, 1 assignment = 1, 2 assignment = 2, 3 assignment = 3
Smoking history	no assignment = 0, yes assignment = 1
Medicate	First-generation EGFR-TKI: gefitinib/icotinib score = 1, Second-generation EGFR-TKI: afatinib score = 2,3rd-generation EGFR-TKI: Ametinib/Osimertinib/Anlotinib Score = 3
Exon 19 deletion	Negative value = 0, positive value = 1
Exon-21 L858R	Negative value = 0, positive value = 1
Exon-20 T790M	Negative value = 0, positive value = 1
Exon-20 S7681	Negative value = 0, positive value = 1
Exon-18 G719X	Negative value = 0, positive value = 1
Exon-21 L861X	Negative value = 0, positive value = 1
SREs	Negative value = 0, positive value = 1
ALP	Enter the original value.
NLR	Enter the original value.
LDH	Enter the original value.
Cyfra21-1	Enter the original value.
CEA	Enter the original value.

**Table 4:** Univariate Cox regression analysis of prognostic OS outcomes.

Indicator	β	SE	Wald χ^2^	*P*	*HR*	95%*CI*	
						Lower limit	Upper limit
Sex	Refer to	Female					
Male	0.018	0.174	0.011	0.917	1.018	0.724	1.433
Age	Refer to	<65					
≧65	1.772	0.508	12.184	<0.001	5.882	2.175	15.907
Exon 19 deletion	Refer to	Negative					
Positive	0.895	0.315	8.094	0.004	2.448	1.321	4.535
Exon-21 L858R	Refer to	Negative					
Positive	1.077	0.303	12.668	<0.001	2.937	1.623	5.316
Exon-20 T790M	Refer to	Negative					
Positive	0.412	0.207	3.964	0.046	1.510	1.006	2.266
NLR	0.062	0.019	10.695	0.001	1.064	1.025	1.104
LDH	0.001	<0.001	6.450	0.011	1.001	1.000	1.001
Cyfra21-1	0.015	0.004	12.726	<0.001	1.015	1.007	1.023
CEA	<0.001	<0.001	7.670	0.006	1.000	1.000	1.001
N-stage	Refer to	N0					
N1	−0.083	0.282	0.087	0.768	0.920	0.529	1.599
N2	0.822	0.317	6.738	0.009	2.275	1.223	4.233
N3	0.083	0.212	0.155	0.694	1.087	0.718	1.646
T-stage	Refer to	T1					
T2	−0.011	0.251	0.002	0.964	0.989	0.605	1.616
T3	0.156	0.277	0.319	0.572	1.169	0.679	2.012
T4	0.041	0.243	0.028	0.866	1.042	0.648	1.676
ECOG rating	Refer to	0					
1	−0.065	0.193	0.111	0.738	0.937	0.642	1.370
2	−0.016	0.390	0.002	0.966	0.984	0.458	2.113
3	−0.160	1.015	0.025	0.875	0.852	0.117	6.230
Smoking history	Refer to	No					
Yes	0.080	0.195	0.167	0.683	1.083	0.739	1.588
Medicate	Refer to	Gefitinib/Icotinib					
Afatinib	−0.260	0.460	0.318	0.573	0.771	0.313	1.900
Amitinib/Osimertinib/Anlotinib Assignment	0.092	0.210	0.190	0.663	1.096	0.726	1.655
SREs	Refer to	Negative					
Positive	0.016	0.315	0.003	0.958	1.017	0.548	1.885
ALP	0.001	0.001	1.455	0.228	1.001	0.999	1.003

#### Multivariate Cox regression analysis of prognostic OS outcomes

In a Cox regression analysis with OS as the dependent variable, independent variables were selected based on their significant differences in univariate Cox regression analysis. These included age, exon 19 deletion, exon-21 L858R, exon-20 T790M, NLR, LDH, Cyfra21-1, CEA, and N-stage. The results showed that age $\geqq$65, higher NLR, higher Cyfra21-1, and higher CEA were independent risk factors associated with OS, affecting patients’ prognosis and survival outcomes. See Table 5.

**Table 5:** Multivariate Cox regression analysis of prognostic OS outcomes.

Indicator	β	*SE*	Wald χ^2^	*P*	*HR*	95%*CI*	
						Lower limit	Upper limit
N-stage	Refer to	N0					
N1	0.027	0.286	0.009	0.923	1.028	0.587	1.801
N2	0.435	0.333	1.705	0.192	1.545	0.804	2.97
N3	−0.203	0.216	0.884	0.347	0.816	0.534	1.247
Age	Refer to	<65					
$\geqq$65	1.355	0.618	4.806	0.028	3.878	1.154	13.024
Exon 19 deletion	Refer to	Negative					
Positive	−0.194	0.362	0.287	0.592	0.824	0.405	1.675
Exon-21 L858R	Refer to	Negative					
Positive	0.464	0.337	1.900	0.168	1.590	0.822	3.077
Exon-20 T790M	Refer to	Negative					
Positive	0.300	0.235	1.639	0.200	1.350	0.853	2.138
NLR	0.059	0.022	6.899	0.009	1.060	1.015	1.108
LDH	<0.001	<0.001	0.564	0.452	1.000	1.000	1.001
Cyfra21-1	0.010	0.005	5.019	0.025	1.011	1.001	1.020
CEA	<0.001	<0.001	5.614	0.018	1.000	1.000	1.001

### Nomogram as a visualisation tool

**Figure 1:**
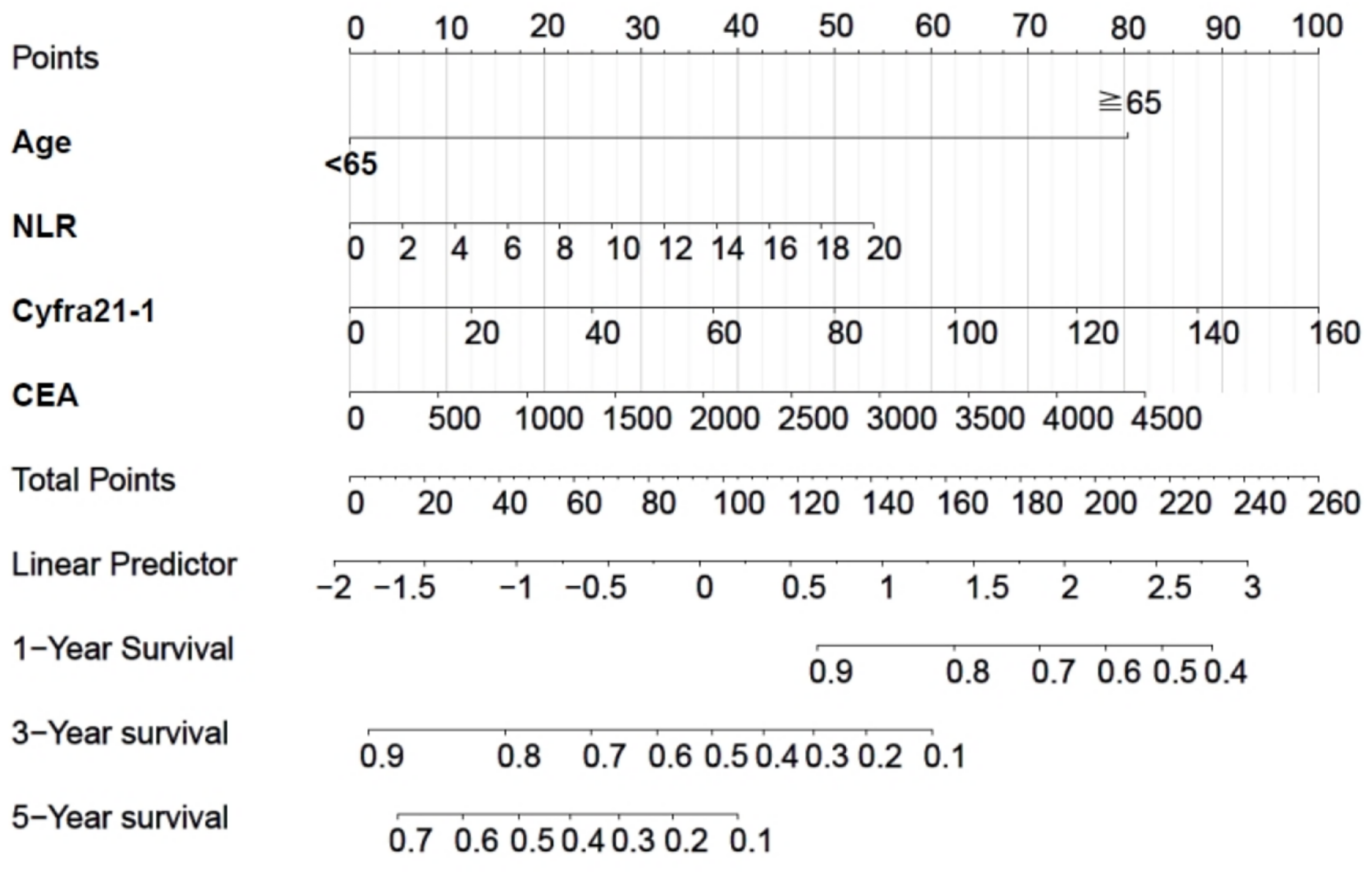
Nomogram of the Cox regression model for the occurrence of OS, a prognostic survival outcome.

To facilitate clinical use, the complex mathematical model was hereby converted into a nomogram (Fig. 1). The scores of the variables included in the model were summed, and a vertical line was then drawn at the total score, which intersected with the three lines representing the predicted OS. The values at the intersection corresponded to the individual predicted 1-, 3-, and 5-year OS.

### Time-ROC analysis of 1, 3, and 5-year prognostic OS models

**Figure 2:**
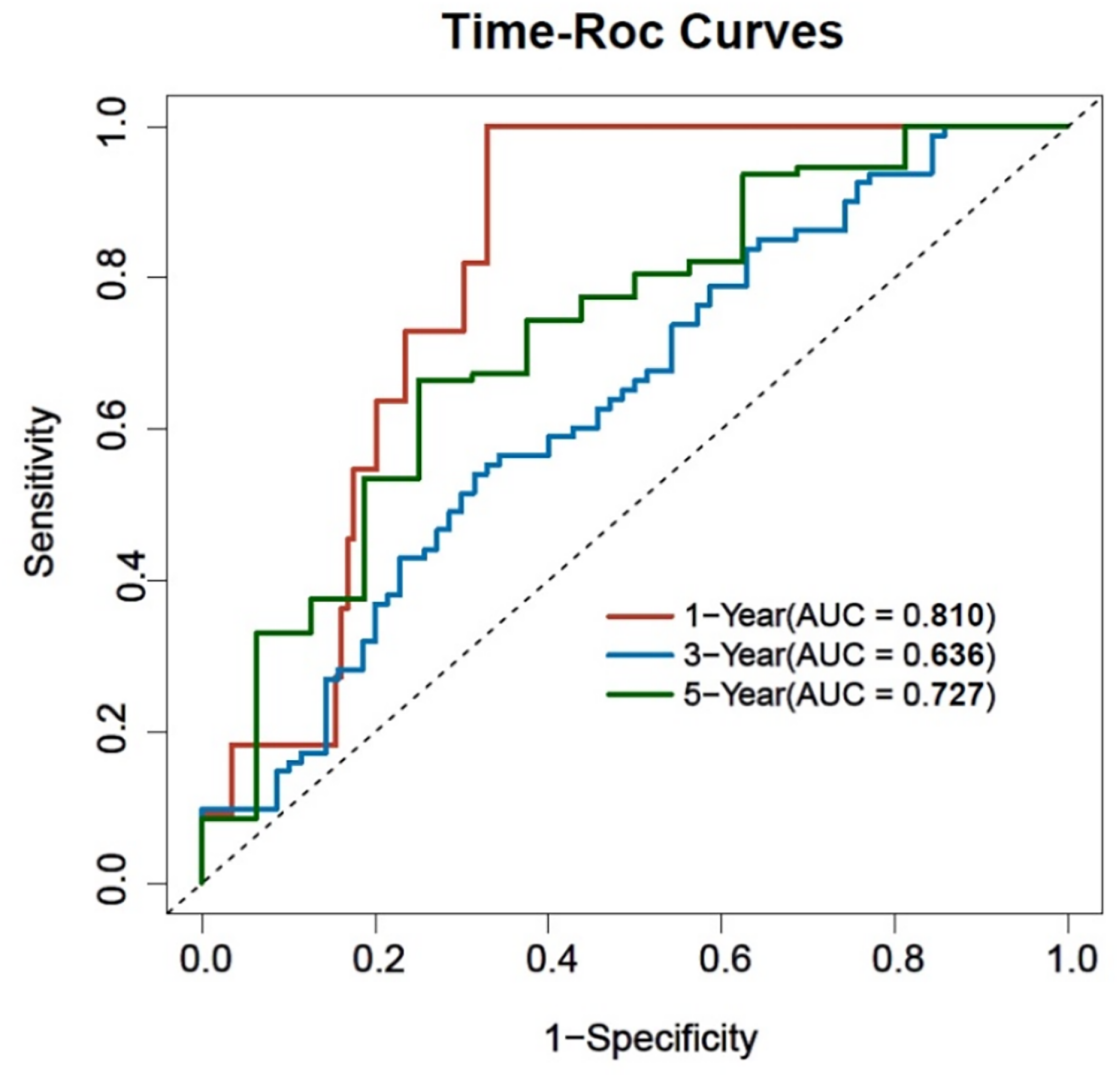
Time-ROC curve for prognostic OS outcomes.

With prognostic OS as the dependent variable and the patient’s multi-factor Cox regression model indicators with differences as the independent variables, a time-ROC curve was plotted. The results showed that the AUC of the model for predicting the occurrence of 1-year OS was 0.810 (0.732, 0.889) (*P* < 0.001), involving a specificity of 0.671 and a sensitivity of 0.998; the AUC for predicting the occurrence of 3-year OS was 0.636 (0.548, 0.725) (*P* < 0.001), exhibiting a specificity of 0.686 and a sensitivity of 0.541; the AUC for predicting the occurrence of 5-year OS was 0.727 (0.591, 0.864) (*P* < 0.001), the specificity was 0.750, and the sensitivity was 0.665. The model demonstrated moderate predictive efficacy, with AUC values exceeding 0.6 at 1, 3, and 5 years. See Table 6 and Fig. 2.

**Table 6:** Time-ROC analysis of 1, 3, and 5-year prognostic OS models.

Time point	AUC	95%CI	*P*	Specificity	Sensitivity
1-year OS rate	0.810	[0.732–0.889]	<0.001	0.671	0.998
3-year OS rate	0.636	[0.548–0.725]	<0.001	0.686	0.541
5-year OS rate	0.727	[0.591–0.864]	<0.001	0.750	0.665

### Calibration testing of 1, 3, and 5-year prognostic OS models

**Figure 3:**
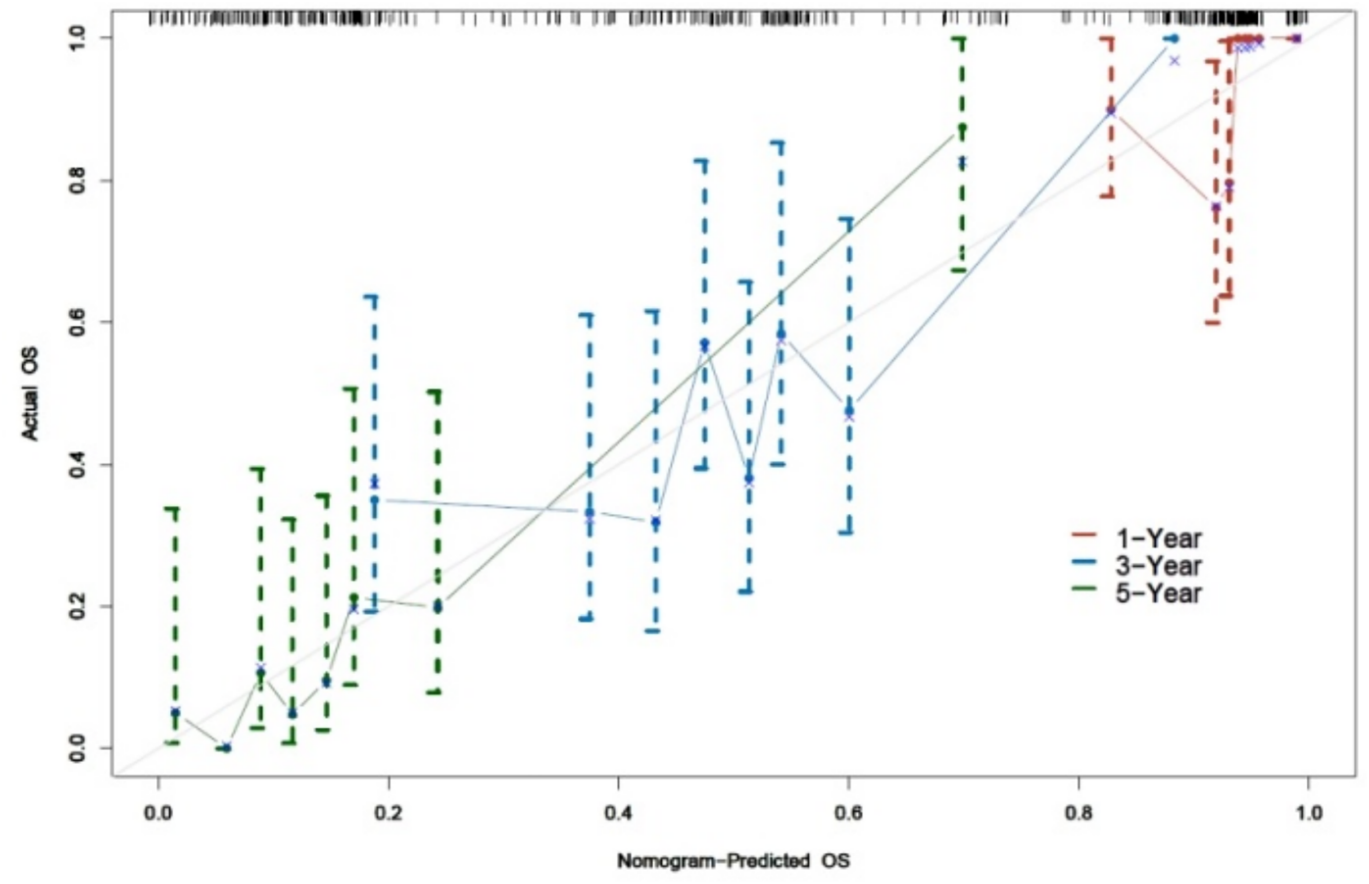
Model calibration plot for the occurrence of OS at 1, 3 and 5 years of prognosis survival outcome.

Verification of the calibration consistency of the model revealed that the C-index of the model was 0.644 (0.590, 0.699) (*P* < 0.001). A C-index >0.6 indicated good consistency of the model. Calibration curve plots uncovered that the calibration curve plots at 1, 3, and 5 years were close to the reference line, indicating high consistency of the model in predicting the occurrence of 1-, 3-, and 5-year survival outcomes OS. See Fig. 3.

### Clinical utility testing of 1, 3, and 5-year prognostic OS models

**Figure 4:**
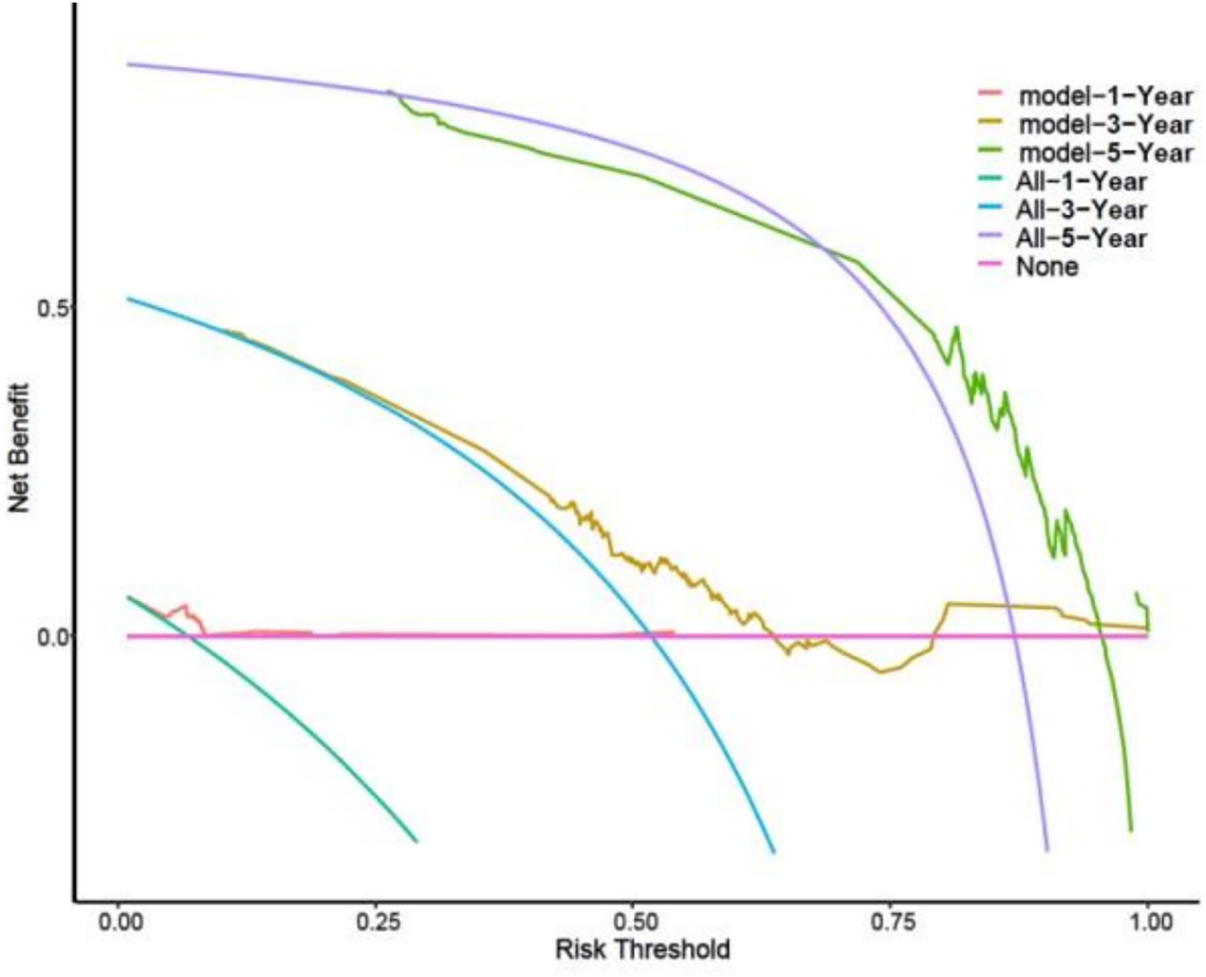
DCA curves of the prognostic model of 1-, 3- and 5-year OS outcomes.

The DCA demonstrated that the model provided a net clinical benefit when the threshold probability of 1-year OS ranged from 5% to 10%, 3-year OS from 25% to 65% and 80% to 100%, and 5-year OS from 70% to 95%. The threshold probability refers to the minimum predicted probability at which a clinician would choose to initiate or adjust a treatment. For example, if a clinician is willing to treat patients whose predicted probability of survival is greater than 25%, the model’s use in this range would provide greater net benefit than either treating all patients or treating none. See Fig. 4.

## Discussion

SREs occur at least once in more than 40% of patients with bone metastases from NSCLC, typically within the first 5 months following diagnosis (Silva et al., 2019). All previous studies regarding the epidemiology of lung cancer have shown a poor prognosis for lung cancer patients with bone metastases (DeSantis et al., 2019; Zhang, Cai & Hong, 2023). The significant variability in survival outcomes attributable to differences in the number and criteria of the included populations underscores the necessity of identifying and specifying prediction factors that affect patients’ prognosis and are important when predicting cancer progression.

Clinical studies have proven the efficiency of EGFR-TKIs in patients with lung cancer bone metastases. Based on the study results, the therapeutic efficacy of gefitinib and erlotinib has been tested on lung adenocarcinoma patients with EGFR mutations, and both medications were found to show 100% efficacy of the tested patients. In particular, the progression of the disease can be delayed, and life quality can be consequently enhanced with the use of EGFR-TKI, representing a positive aspect of bone metastasis in OS patients. Besides, osimertinib has been shown to be effective in the treatment of lung cancer bone metastases patients in clinical trials (Lin et al., 2015; Gen et al., 2022). Additionally, we examined the treatment context of EGFR-TKI administration to better interpret the prognostic model. Our data showed that although EGFR-TKIs were used as the first-line treatment, over one-fifth of patients had received chemotherapy, radiotherapy, or surgery prior to initiation, and more than half received additional treatments afterward. This highlights the complex and evolving nature of clinical management in this patient population. Early discontinuation occurred in approximately 9% of cases, and treatment changes such as switching to osimertinib or combining with anlotinib were recorded in 17 patients. These findings reflect both clinical adaptation to disease progression and patient-specific therapeutic needs. Inclusion of such information in our analysis ensures a more accurate contextual understanding of the nomogram’s predictive performance and its applicability to real-world practice. Furthermore, dose information was available in 36 patients. The most recorded regimens included gefitinib 250 mg QD, icotinib 125 mg TID, and afatinib 30 mg QD, which was consistent with standard treatment protocols. Though explicit documentation of dose modifications was infrequent, the available data suggest that most patients adhered to standard dosing schedules throughout the course of EGFR-TKI therapy. These data support the assumption of treatment consistency underlying our model’s construction and interpretation.

On one hand, we observed that the levels of ALP, NLR, LDH, Cyfra21-1 and CEA in the blood decreased significantly after EGFR-TKI treatment (*P* < 0.05) compared to the baseline. This might reflect a favourable treatment response and effective disease control, potentially resulting from the treatment’s impact on inflammatory activity or bone metabolism. For example, a reduction in NLR may indicate less inflammation, thus supporting the conclusion that chemotherapy could lead to enhanced NLR and reduce the body’s response to NLR (Ozturk et al., 2024). Furthermore, the study also saw an increase in the rate of bone-related disorders in patients following therapy (*P* < 0.05). The observed decrease in serum markers may be misinterpreted as an effect of treatment; however, it is likely due to the confluence of multiple factors during treatment. This situation may arise from the delayed response of local bone metastases to treatment, or from the complex interplay between the bone microenvironment and tumour microenvironment. Notably, the small sample size may limit the generalisability and statistical significance of the results, while individual differences among participants (*e.g*., underlying diseases, treatment response) may also affect the comparability of the results. Additionally, as this was a single-center study, potential selection bias cannot be ruled out, which may limit the generalizability of our findings to broader populations. To further validate these findings, future studies should consider conducting multicenter, large-scale studies to improve the external validity and generalisability of the results.

This study included 164 patients, and multiple variables were screened using Cox regression analysis to construct a prognostic nomogram model. Although EGFR exon 19 deletion was significant in univariate analysis, it lost significance in the multivariate model. This may be due to collinearity with other prognostic factors, such as systemic inflammatory markers and nutritional status, which were also closely associated with survival. Additionally, since all patients received EGFR-TKI therapy, the therapeutic effect may have been relatively uniform across mutation subtypes, thereby attenuating the prognostic distinction in multivariate modeling. The model included four clinical indicators, including an inflammatory response marker (NLR), tumour burden indicators (Cyfra21-1 and CEA), and demographic data (age). The joint assessment of these variables could facilitate to more comprehensively reveal the biological characteristics of the tumour and predict a patient’s response to EGFR-TKI therapy. The model assigned a score to each variable, and these scores were further summed to generate a total score. A vertical line was drawn from the patient’s point to intersect with the three survival curves representing 1-year, 3-year, and 5-year OS. The intersection points on these curves corresponded to the predicted survival times for the respective time intervals. The Nomogram predicted the clinical outcomes of the patients and specifically identified the subset of patients with a high probability of death while receiving EGFR-TKI treatment. For these high-risk patients, clinicians may consider the less frequent bias among the risk factors and modify the treatment plan by combining them with immunotherapy or chemotherapy to improve treatment efficacy and enhance patient survival. By contrast, overtreatment may be avoided among low-risk patients, thereby ensuring both decrease of unnecessary side effects and the enhancement of life of the patient. While our model demonstrated acceptable discrimination at 1- and 5-year time points (AUCs of 0.810 and 0.727, respectively), its performance at 3 years was more modest (AUC = 0.636). Similarly, the C-index of 0.644 suggests moderate overall discriminative ability. These findings indicate that, although the model may aid in stratifying patients by risk, especially in the short term, caution is warranted when applying it to guide intermediate-term prognosis. Further refinement and external validation are needed to enhance its clinical applicability.

Models can provide important support for clinical decision-making, especially when assessing a patient’s survival risk at different time points. These threshold intervals can guide clinicians in stratifying patients for different levels of intervention. For instance, patients with low predicted probability of 1-year OS (<10%) might be considered for best supportive care or enrollment in clinical trials. In contrast, patients with high predicted 3- or 5-year OS (*e.g*., >70%) may benefit from continued EGFR-TKI monotherapy or less intensive monitoring. Intermediate ranges (*e.g*., 25–65% for 3-year OS) could indicate a need for more aggressive combination therapies, closer surveillance, or evaluation for second-line options, depending on clinical context. Thus, the model not only facilitates short-term prognostication, but also forges a basis for long-term treatment decisions, thereby maximizing clinical benefits. While the model demonstrates high utility in predicting OS, limitations persist. First, the model should be further validated in different patient groups and disease types. Second, the prediction accuracy may be compromised for patients with multiple comorbidities or those who are elderly and frail, necessitating further optimization. Overall, the model provides effective support for personalized treatment plans, and future studies should focus on its validation and optimization.

Serum marker testing boasts many advantages, including its non-invasiveness and the ability to obtain fast and easily reproducible measurements. However, it is not sufficient to provide a quick and comprehensive picture of the situation. This study offers novel insights by jointly evaluating the prognostic value of serum markers in patients with lung adenocarcinoma and bone metastases treated with EGFR-TKI. First, the results show that the levels of serum markers such as NLR, CEA and Cyfra21-1 are significantly correlated with patients’ survival and treatment response. Additionally, fluctuations in serum marker levels before and after EGFR-TKI treatment can provide valuable information for clinicians when devising treatment plans. In lung adenocarcinoma cases, a rise in these markers typically indicates an increase in tumour burden as well as disease progression. It has been shown that higher levels of this oncogene are significantly associated with lower PFS and OS when correlated with transporter genes (CEA and Cyfra 21-1). This association may serve as an important predictor for erlotinib treatment in patients with advanced NSCLC (Fiala et al., 2014). Notably, serum markers for cancer represent only one aspect of the broader diagnostic picture. Several studies have indicated that patients with a high NLR face significantly higher frequencies of metastasized organs such as brain, liver, and bone compared to those with a low NLR. Additionally, baseline NLR levels serve as an independent prognostic factor in patients with EGFR-mutant NSCLC (Aguiar-Bujanda et al., 2018; Chen et al., 2023). Li et al. (2023) demonstrated that higher CYFRA 21-1 levels may lead to worse OS and PFS, and recognized it as a significant predictor in patients with wild-type EGFR NSCLC.

Limitations of this study include its small sample size and that it was a retrospective study, which restricts the general applicability of the results. In addition, the collection of all potential confounding factors, such as the patient’s lifestyle, comorbidities, and detailed treatment information, is not feasible, which may affect the prognosis. Therefore, future studies should involve larger prospective studies to validate the present findings.

## Conclusion

This study evaluated the prognostic predictive value of the combined application of serum markers (NLR, CEA, and Cyfra21-1) in patients with lung adenocarcinoma bone metastases receiving EGFR-TKI therapy. A nomogram for predicting prognosis was constructed using Cox regression analysis. This tool identifies patients who may respond poorly to EGFR-TKI treatment, providing a basis for personalized treatment, optimising treatment plans, and improving the prognosis. The study results further highlight the potential of integrating multiple biomarkers to improve the accuracy of treatment response prediction and inform treatment strategy adjustments, thereby advancing precision medicine and maximizing therapeutic outcomes.

## Supplemental Information

10.7717/peerj.20537/supp-1 Supplemental Information 1 Initial raw data.

10.7717/peerj.20537/supp-2 Supplemental Information 2 Division table.

## Additional Information and Declarations

### Data Availability

The following information was supplied regarding data availability:

The raw data is available in the Supplemental File.
