# Supplementary material for: Serum markers for prognostic value of EGFR-TKI in lung adenocarcinoma with bone metastases: a retrospective study
Source: PeerJ. 2026 Jan 14;14:e20537. doi: 10.7717/peerj.20537 (PMC12811968; doi:10.7717/peerj.20537)
Supplement: Supplemental Information 2 [file peerj-14-20537-s002.docx]

Division Table

| variable | assignnt method |
| --- | --- |
| sex | Female value = 0, male value = 1 |
| age | <65 years old is assigned a value of 0, ≧65 years old is assigned a value of 1 |
| T-stage | T1 is assigned the value 1, T2 is assigned the value 2, T3 is assigned the value 3, and T4 is assigned the value 4. |
| N-stage | N0 is assigned the value 0, N1 is assigned the value 1, N2 is assigned the value 2, and N3 is assigned the value 3. |
| ECOG rating | 0 assignment = 0, 1 assignment = 1, 2 assignment = 2, 3 assignment = 3 |
| Smoking history | no assignment = 0, yes assignment = 1 |
| medicate | First-generation EGFR-TKI: gefitinib/icotinib score = 1,Second-generation EGFR-TKI: afatinib score = 2,3rd-generation EGFR-TKI: Ametinib/Osimertinib/Anlotinib Score = 3 |
| exon 19 deletion | Negative value = 0, positive value = 1 |
| exon-21 L858R | Negative value = 0, positive value = 1 |
| exon-20 T790M | Negative value = 0, positive value = 1 |
| exon-20 S7681 | Negative value = 0, positive value = 1 |
| exon-18 G719X | Negative value = 0, positive value = 1 |
| exon-21 L861X | Negative value = 0, positive value = 1 |
| SREs | Negative value = 0, positive value = 1 |
